# Eosinophils may play regionally disparate roles in influencing IgA^+^ plasma cell numbers during large and small intestinal inflammation

**DOI:** 10.1186/s12865-016-0153-0

**Published:** 2016-05-31

**Authors:** Ruth Forman, Michael Bramhall, Larisa Logunova, Marcus Svensson-Frej, Sheena M. Cruickshank, Kathryn J. Else

**Affiliations:** Department of Immunology, University of Manchester, Manchester, UK; Immunology section, Lund University, BMC D14, Lund, SE-22184 Sweden

**Keywords:** Eosinophil, Plasma cell, B cell, Region, Intestinal, Inflammation, Trichuris, Toxoplasma

## Abstract

**Background:**

Eosinophils are innate immune cells present in the intestine during steady state conditions. An intestinal eosinophilia is a hallmark of many infections and an accumulation of eosinophils is also observed in the intestine during inflammatory disorders. Classically the function of eosinophils has been associated with tissue destruction, due to the release of cytotoxic granule contents. However, recent evidence has demonstrated that the eosinophil plays a more diverse role in the immune system than previously acknowledged, including shaping adaptive immune responses and providing plasma cell survival factors during the steady state. Importantly, it is known that there are regional differences in the underlying immunology of the small and large intestine, but whether there are differences in context of the intestinal eosinophil in the steady state or inflammation is not known.

**Results:**

Our data demonstrates that there are fewer IgA^+^ plasma cells in the small intestine of eosinophil-deficient ΔdblGATA-1 mice compared to eosinophil-sufficient wild-type mice, with the difference becoming significant post-infection with *Toxoplasma gondii*. Remarkably, and in complete contrast, the absence of eosinophils in the inflamed large intestine does not impact on IgA^+^ cell numbers during steady state, and is associated with a significant increase in IgA^+^ cells post-infection with *Trichuris muris* compared to wild-type mice. Thus, the intestinal eosinophil appears to be less important in sustaining the IgA^+^ cell pool in the large intestine compared to the small intestine, and in fact, our data suggests eosinophils play an inhibitory role. The dichotomy in the influence of the eosinophil over small and large intestinal IgA^+^ cells did not depend on differences in plasma cell growth factors, recruitment potential or proliferation within the different regions of the gastrointestinal tract (GIT).

**Conclusions:**

We demonstrate for the first time that there are regional differences in the requirement of eosinophils for maintaining IgA+ cells between the large and small intestine, which are more pronounced during inflammation. This is an important step towards further delineation of the enigmatic functions of gut-resident eosinophils.

**Electronic supplementary material:**

The online version of this article (doi:10.1186/s12865-016-0153-0) contains supplementary material, which is available to authorized users.

## Background

Eosinophils were originally discovered based on their distinctive “eosin-loving” intracellular granules. These granules contain hydrolytic enzymes and pre-formed cationic granule proteins, including major basic protein (MBP), eosinophil cationic protein (ECP), eosinophil peroxidase (EPO) and eosinophil-derived neurotoxin (EDN). Eosinophils have long been described as end-stage effector cells acting through the secretion of granule-derived proteins, which exert toxic effects on parasites and microbes, but can also cause collateral damage to host tissue cells, especially in allergic inflammation (reviewed [1, 2]). More recently eosinophils have been described as multi-functional leukocytes, acting as sources of numerous cytokines, chemokines, matrix metalloproteinases and reactive oxygen species with a range of functions (reviewed [3]), in addition to production of eosinophil-specific mediators. Importantly, many, if not all, of the effector molecules are stored within eosinophil-specific granules, allowing for very rapid secretion without the need for *de novo* synthesis [4]. Alongside the increasing repertoire of eosinophil-derived products there has been an increasing awareness of the broader role eosinophils play in immunity, with a plethora of roles identified for them, including helping shape adaptive immune responses and providing plasma cell survival factors in the bone marrow [5, 6].

Under steady state conditions the gastrointestinal tract (GIT) contains the largest number of eosinophils in the body [7, 8]. Intestinal eosinophils reside primarily in the lamina propria and are important in the maintenance of immune homeostasis in gut-associated tissues [9]. Although the GIT is often considered as a single entity, the large and small intestine are anatomically and functionally different and therefore should be analysed as two separate immunological compartments [10]. In the small intestine there is a higher frequency of eosinophils than in the large intestine [11] and the eosinophil populations in the large and small intestine are phenotypically different [12]. The functional significance of these phenotypic variants is however not known, although the increased frequency of eosinophils in the small versus large intestine implies they may be of greater functional significance in this region of the GIT, at least in the steady state.

Despite the literature describing differences in the number and phenotype of eosinophils in the naïve small and large intestine, and a functional role for the eosinophil in supporting plasma cells during steady state conditions, it is not known whether the small intestinal eosinophil has unique functions compared to the large intestinal eosinophil and whether this is altered during inflammation. Eosinophilia is observed in response to infection and during inflammation of both the large [13, 14] and small intestine [15], and virtually any inflammatory condition of the GIT can feature an eosinophilia. Thus, eosinophils are not simply indicative of a Th2 disorder, but rather can be prominent in many diverse inflammatory conditions. Indeed, a number of human and translational studies have shown that eosinophils are increased in intestinal tissues affected by inflammatory bowel disease [14]. Here we use two models of parasitic infection – chronic *Trichuris muris* [16] infection and *Toxoplasma gondii* infection, that drive an inflammatory response in the GIT restricted to the large and small intestine, respectively. Thus use of these two complementary infection models allows a dissection of the functional roles of the eosinophil in the context of the IgA^+^ cells in both the large intestine and small intestine.

## Results

### *T. muris* and *T. gondii* infections drive eosinophilia in the large and small intestine

**Fig. 1:**
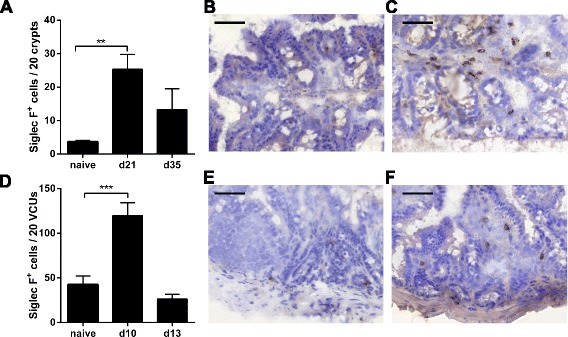
Siglec F expression post-infection. **a**; Quantification of Siglec F^+^ cells from BALB/c naïve mice (*n* = 7) and at 21 (*n* = 4) and 35 (*n* = 5) days and post-infection with *T. muris*. Representative photos of immunohistochemistry in large intestine tissue from BALB/c (**b**); naïve and (**c**); at 21 days post-infection with *T. muris* (*n* = 4). **d**; Quantification of Siglec F^+^ cells from BALB/c naïve mice (*n* = 6) and at 10 (*n* = 5) and 13 (*n* = 4) days post-infection with *T. gondii* PRU . Representative photos of immunohistochemistry in small intestine tissue from BALB/c (**e**); naïve and (**f**); at 10 days post-infection with *T. gondii* PRU.*p < 0.05, **p < 0.01, ***p < 0.001 student’s *t*-test. Scale Bar represents 50 μm

At day 21 and 35 following a low dose (20 egg) *T. muris* infection, we quantified large intestine eosinophilia and analysed eosinophil distribution using immunohistochemical staining with the eosinophil-specific marker Siglec-F [17]. A significant intestinal eosinophilia was observed in wild-type mice, with an influx of eosinophils primarily into the lamina propria of the large intestine evident at day 21 post-infection, subsiding back to naïve levels by d35 post-infection (Fig. 1a–c; One-way ANOVA F (2,13) = 7.835, *p* = 0.0059 with a post-hoc Dunnett’s test showing an effect at d21 compared to naïve (*p* < 0.01)). To determine if the same trend was also observed in the small intestine post-infection, mice were orally infected with 1 million *T. gondii* tachyzoites, using a Type II strain (Pruginaud). Infection with *T. gondii* also resulted in a significant eosinophilia, this time in the small intestine at d10 post-infection, returning to naïve levels by d13 post-infection, and with eosinophils residing primarily in the lamina propria (Fig. 1d–f; One-way ANOVA *F* (2,12) = 19.83, *p* = 0.0002 with a post-hoc Dunnett’s test showing an effect at d10 compared to naïve (*p* < 0.001)).

### IgA expression during *T. gondii* infection

**Fig. 2:**
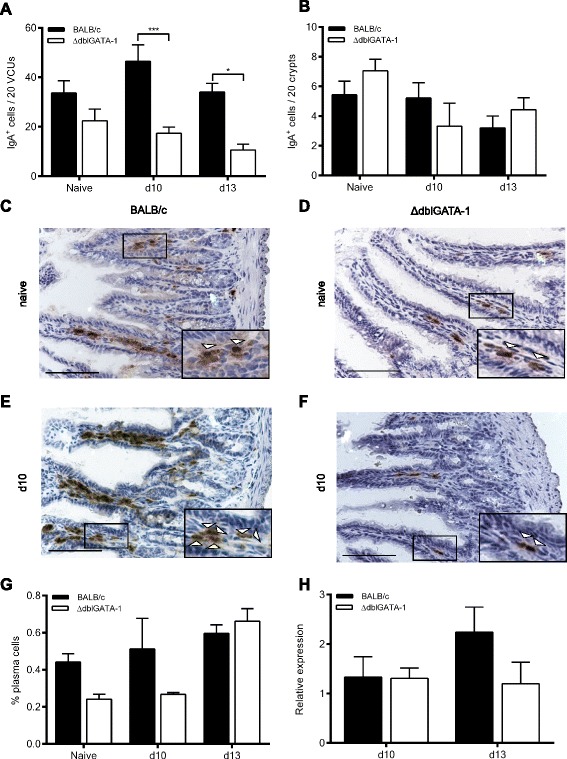
Expression levels of IgA in the gastrointestinal tract of mice post infection with *T. gondii* PRU. Quantification of IgA^+^ cells in **a**: small intestine and **b**: large intestine of BALB/c and ΔdblGATA-1 mice following infection with *T. gondii* PRU. BALB/c and ΔdblGATA-1 naïve mice (*n* = 6; combined from 2 independent experiments), 10 days post-infection (*n* = 7-9; combined from 2 independent experiments) and 13 days post-infection (*n* = 4). **c**-**f**. representative photos of immunohistochemistry in naïve and at day 10 post infection in small intestine tissue from BALB/c and ΔdblGATA-1 mice (**g**). Quantification of CD138^+^B220^lo^ plasma cells in the mesenteric lymph nodes of naïve and infected mice (*n* = 3-5) and (**h**). brain parasite burden expressed as relative expression of bradyzoite amplification compared to murine housekeeping gene *p < 0.05, **p < 0.01, ***p < 0.001. Two-way ANOVA with post-hoc Bonferroni Scale Bar represents 100 μm. Arrowheads in inset images indicate positive cells

To determine the effect of eosinophil-deficiency on plasma IgA^+^ cell numbers post-infection, ΔdblGATA-1 and wild-type mice on a BALB/c background were infected with *T. gondii*. ΔdblGATA-1 mice harbour a deletion within the *Gata1* promoter, which prevents the development of mature eosinophils [18]. IgA^+^ cells in the GIT sections were quantified by immunohistochemistry, staining with an anti-IgA antibody which has been described as a marker of gut plasma cells [19]. In the small intestine ΔdblGATA-1 mice had a non-significant decrease in the number of IgA^+^ cells in steady state, compared to BALB/c mice, which reached significance following *T. gondii* infection (Fig. 2a, c–f; Two-way ANOVA showed a significant effect of genotype *F* (1,30) = 28.72, *p* < 0.001 with a post-hoc Bonferonni test showing a significant effect at d10 (*p* < 0.0001) and d13 (*p* < 0.05) post-infection), although there was no significant increases in IgA^+^ cell numbers in mice of either genotype post-infection. In contrast, in the large intestine, which is not exposed to the parasite, no significant changes in IgA^+^ cell numbers were observed post-infection or in the absence of eosinophils (Fig. 2b). Additionally, plasma cells, defined as B220^lo^CD138^+^, were analysed in mesenteric lymph nodes (MLN). No significant changes were observed post-infection or in the absence of eosinophils (Fig. 2g). Importantly, ΔdblGATA-1 and wild-type mice harboured similar parasite burdens, with no significant differences in brain cysts at the time points analysed (Fig. 2h); this allows the parasite to be used purely as a driver of inflammation without the addition of any confounding factor.

### IgA expression during *T. muris* infection

**Fig. 3:**
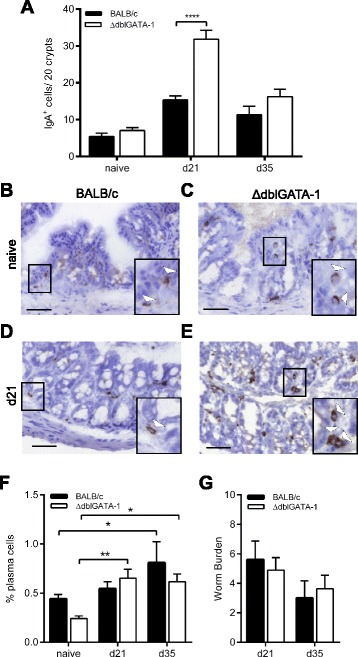
Expression levels of IgA in the gastrointestinal tract of mice post *T. muris* infection. **a**; Quantification of IgA^+^ cells from BALB/c and ΔdblGATA-1 naïve mice (*n* = 6-7; combined from 2 independent experiments), at 21 days post-infection (*n* = 12; combined from 3 independent experiments) and 35 days post-infection (*n* = 3) and (**b**-**e**); representative photos of immunohistochemistry in large intestine tissue in naïve mice and at d21 post infection. **f** Quantification of CD138^+^B220^lo^ cells in the mesenteric lymph nodes of naïve and infected mice (*n* = 3-7) and (**g**). *T. muris* worm burden in the ceacum and large intestine of infected mice (*n* = 8-11, combined from 2 independent experiments *p < 0.05, **p < 0.01, ***p < 0.001. Two-way ANOVA with post-hoc Bonferroni. Scale Bar represents 100 μm. Arrowheads in inset images indicate positive cells

Although IgA^+^ plasma cells numbers was assessed in the large intestine following *T. gondii* PRU infection, the infection does not reach the large intestine. We therefore chose to analyse large intestinal IgA^+^ cell numbers during inflammation driven by a *T. muris* low dose infection of ΔdblGATA-1 and wild-type mice on a BALB/c background. *T. muris* specifically infects the caecum and proximal colon of mice and drives a large-intestinal inflammatory response. In naïve mice we detected no significant differences in IgA^+^ cell numbers between BALB/c and ΔdblGATA-1 mice (Fig. 3a). However, we observed an increase in IgA^+^ cells in the large intestine of both ΔdblGATA-1 mice and BALB/c mice post-infection (Fig. 3a–e) (Two-way ANOVA showed a significant effect of infection *F* (2,42) = 63.79, *p* < 0.0001 with a post-hoc Bonferonni test showing a significant effect at d21 (*p* < 0.0001, BALB/c and ΔdblGATA-1 mice) and d35 (*p* < 0.05 ΔdblGATA-1 mice; *p* = NS BALB/c). Moreover as well as changes post-infection, an unanticipated significant effect of genotype was observed with significantly higher numbers of IgA^+^ plasma cells in the ΔdblGATA-1 mice (*F* (1,42) = 17.51, *p* = 0.0001 with a post-hoc Bonferonni test showing a significant effect (*p* < 0.0001) at d21 post-infection). Flow cytometry analysis of MLN cells demonstrated no significant effect of eosinophil-deficiency on plasma cells under steady state conditions, or at d21 or day 35 post-infection, suggesting that the observed changes were only local to the site of infection. However, there was a significant increase in plasma cells detected post-infection (Fig. 3f) (effect of time *F* (2,29) = 7.819, *p* = 0.0019 with a post-hoc Bonferonni test showing significance compared to naïve at d35 in BALB/c mice (*p* < 0.05), and at d21 (*p* < 0.01) and d35 (*p* < 0.05) post-infection in ΔdblGATA-1 mice). Comparably to the *T. gondii* infection, analysis of worm burden at d21 and d35 post-infection showed no significant differences between ΔdblGATA-1 and wild-type mice (Fig. 3g), allowing the infection to be used purely as a driver of inflammation without any confounding factors.

### IgA expression during *T. gondii* RH infection

**Fig. 4:**
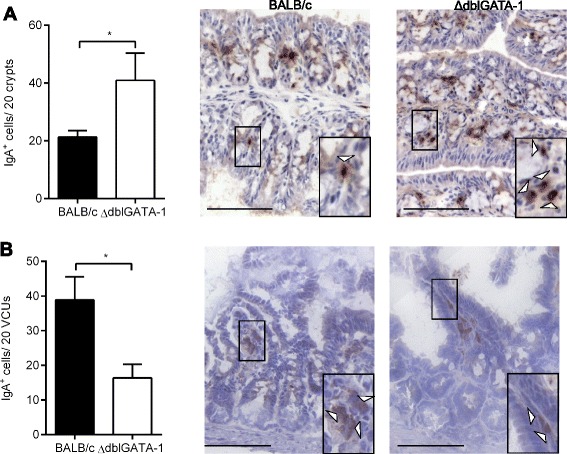
Expression levels of IgA in the gastrointestinal tract of mice post infection with *T. gondii* RH. **a**; Quantification of IgA expressing cells in large intestine tissue from BALB/c and ΔdblGATA-1 naïve mice and at day 5 post infection with *T. gondii* RH (*n* = 6-9; combined from 2 independent experiments). **b**; Quantification of IgA expressing cells in small intestine tissue from BALB/c and ΔdblGATA-1 naïve mice and at day 5 post infection with *T. gondii* RH (*n* = 6-9; combined from 2 independent experiments). *p < 0.05, **p < 0.01, ***p < 0.001. Student’s *t*-test

Although both types of infection utilised (chronic *T. muris* and PRU strain *T. gondii*) drive intestinal inflammation in mice, *T. muris* is a helminth whereas *T. gondii* is an intracellular protozoan parasite. In order to remove this confounding factor, mice were infected with the RH strain of *T. gondii* and the IgA^+^ plasma cell phenotype analysed in the two areas of the GIT. The RH type I strain of *T. gondii* is more virulent than the type II PRU strain, with the parasite reaching and causing inflammation in both the small and large intestine (unpublished observations). Analyses of ΔdblGATA-1 and BALB/c mice infected with RH demonstrated that the changes in IgA^+^ plasma cell numbers were independent of the type of parasitic infection. Thus, consistent with previous observations, significantly more IgA^+^ cells (Fig. 4a; *p* = 0.0302, Student’s *t*-test) were present in the large intestine, while significantly fewer IgA^+^ cells (Fig. 4b; *p* = 0.0239, Student’s *t*-test) were detected in the small intestine in ΔdblGATA-1 mice compared to BALB/c mice post-infection.

Having confirmed regional differences in the importance of the eosinophil in sustaining IgA^+^ plasma cells using the same parasitic infection (*T. gondii* RH), all subsequent experiments utilised low dose *T. muris* infections for analyses of the large intestine and *T. gondii* PRU infections for analyses of the small intestine, respectively. Although infection with *T. gondii* RH allowed simultaneous analysis of both the small and large intestine, the more virulent infection was associated with higher morbidity and therefore not utilised in further experiments.

### Plasma cell survival, recruitment factors and proliferation during *T. muris* and *T. gondii* infection

**Fig. 5:**
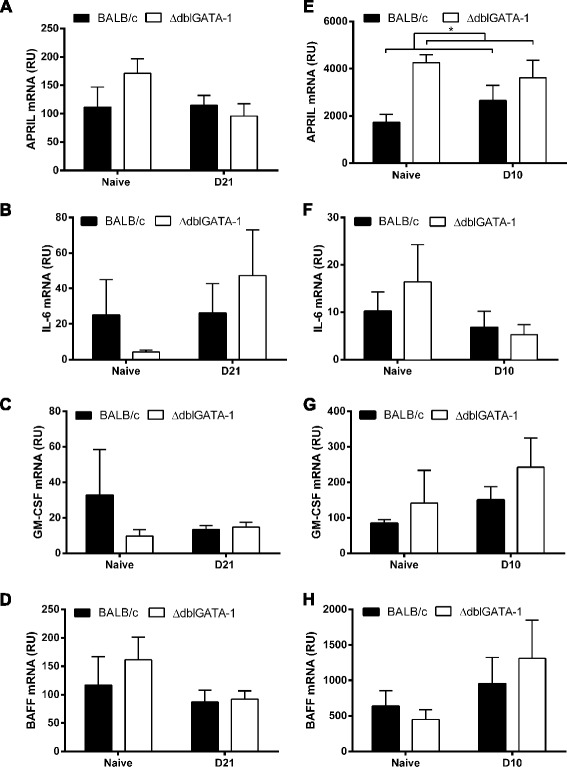
Involvement of plasma cell survival factors in differences in gastrointetstinal tract plasma cell numbers. **a**; APRIL **b**; IL-6 **c**; GM-CSF and **d**; BAFF mRNA expression (relative units, RU) was assessed by qPCR in samples of large intestine tissue from BALB/c and ΔdblGATA-1 mice. Data shows naïve mice (*n* = 4) and at day 21 post-infection with *T. muris* (*n* = 6-9; combined from 2 independent experiments). **e**; APRIL **f**; IL-6 **G**; GM-CSF and **h**; BAFF mRNA expression in small intestine tissue from BALB/c and ΔdblGATA-1 mice; naïve mice (*n* = 2-4) and a day 10 post-infection with *T. gondii* PRU (*n* = 4-8 combined from 2 independent experiments) *p < 0.05 2-way ANOVA, effect of genotype

As survival factors derived from eosinophils are important in the maintenance of plasma cells in the bone marrow [5], we analysed production of these factors in the small and large intestine of naïve and infected mice. Analyses of APRIL, IL-6, GM-CSF and BAFF by qPCR in the large intestine of naïve BALB/c and ΔdblGATA-1 mice and at d21 post-infection with *T. muris* revealed no significant differences in levels of any of the survival factors (Fig. 5a-d). Analyses of small intestine naïve tissue or d10 post-infection with *T. gondii* revealed an increase in APRIL mRNA in ΔdblGATA-1 mice (Two-way ANOVA showed a significant effect of genotype *F* (1,19) = 6.086, *p* = 0.0233, post-hoc Bonferonni tests were non-significant), but there were no significant differences in any other survival factors (Fig. 5e–h). The increase in APRIL expression in the small intestine of ΔdblGATA-1 mice is unlikely to underlie the decreased plasma cells observed in the small intestine of these mice as, if anything, an increased number of plasma cells would be expected.

**Fig. 6:**
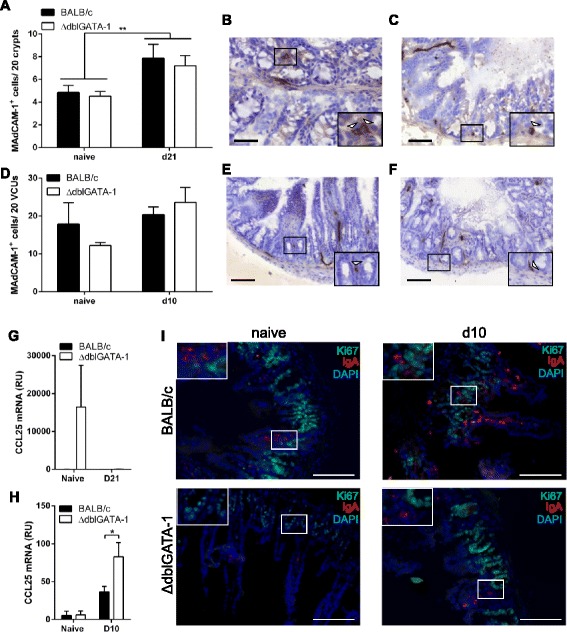
Involvement of recruitment factors in gastrointestinal tract plasma cells. **a** Quantification of MAdCAM-1 protein expression in large intestine tissue from BALB/c and ΔdblGATA-1 naïve mice (*n* = 7-8; combined from 2 independent experiments) and at 21 days post-infection with *T. muris* (*n* = 6-9; combined from 2 independent experiments). Representative photos of immunohistochemistry in large intestine tissue from (**b**); BALB/c and (**c**); ΔdblGATA-1 mice at 21 days post-infection with *T. muris.* **d**; Quantification of MAdCAM-1 protein expression in small intestine tissue from BALB/c and ΔdblGATA-1 naïve mice (*n* = 4-6; combined from 2 independent experiments) and at day 10 post infection with *T. gondii* PRU (*n* = 5-8; combined from 2 independent experiments). Representative photos of immunohistochemistry in small intestine tissue from (**e**); BALB/c and (**f**); ΔdblGATA-1 mice at 10 days post-infection with *T. gondii* PRU. **g**; CCL25 mRNA expression was assessed by Q-PCR in samples of large intestine tissue from BALB/c and ΔdblGATA-1 mice. Data shows naïve mice (*n* = 3-4) and at day 21 post-infection with *T. muris* (*n* = 6-8; combined from 2 independent experiments). **h**; CCL25 mRNA expression in small intestine tissue from BALB/c and ΔdblGATA-1 mice; naïve mice (*n* = 4-5) and at day 10 post-infection with *T. gondii* PRU (*n* = 8; combined from 2 independent experiments). *p < 0.05, **p < 0.01, ***p < 0.001. Two-way ANOVA with post-hoc Bonferroni or student’s *t*-test as appropriate. Scale Bar represents 50 μm. **i**; Representative photos of immunohistochemistry in the small intestine tissue from BALB/c and ΔdblGATA-1 mice: naïve and at 10 days post-infection with *T. gondii* PRU. Sections are stained with IgA (red), Ki67 (green) and DAPI (blue). Scale Bar represents 100 μm

As we observed no change in plasma cell survival factors that correlated with the regional changes in IgA^+^ plasma cell numbers observed in eosinophil-deficient mice, we investigated whether a change in plasma cell recruitment post-infection in the absence of eosinophils, might underlie the marked changes in plasma cell numbers. Both MAdCAM-1 [19] and CCL25 [20] are important in recruitment of plasma cells to the intestine. Following infection with *T. muris*, a significant increase in the number of MAdCAM-1 expressing cells was observed in the large intestine of both ΔdblGATA-1 and BALB/c mice (Fig. 6a-c; Two-way ANOVA showed a significant effect of infection *F* (1,26) = 9.413, *p* = 0.0050). In contrast, following infection with *T. gondii* no significant changes in MAdCAM-1 expression was observed in the small intestine (Fig. 6d–f; Two-way ANOVA, effect of infection *p* = NS). Importantly, we saw no difference in MAdCAM-1 protein expression between ΔdblGATA-1 and BALB/c mice in either the naïve or infected mice in the small or large intestine following infection with *T. gondii* or *T. muris* respectively (Fig. 6a–f) (Two-way ANOVA, effect of genotype *p* = NS). Moreover, no significant differences in CCL25 message were found in the large intestine in either naïve mice or following infection with *T. muris*, in the presence or absence of eosinophils (Fig. 6g). In contrast, in the small intestine there was an increase in CCL25 message following infection (Fig. 6h; Two-way ANOVA, significant effect of infection *F* (1,19) = 12.62, *p* = 0.0021; effect of genotype *p* = NS), with a significant increase detected at d10 post-infection between ΔdblGATA-1 and control mice (post-hoc Bonferroni test *p* < 0.05). Given that the number of plasma cells in the small intestine post-*T. gondii* infection was significantly lower in the absence of eosinophils, the importance of the upregulation of CCL25 in the small intestine of ΔdblGATA-1 mice is unclear, but may potentially reflect a compensatory mechanism. Collectively, our data suggests that eosinophil-dependent regulation of MAdCAM-1 or CCL25 does not underlie the reduction in plasma cell numbers in the small intestine and elevated plasma cell numbers in the large intestine observed in the ΔdblGATA-1 compared to wild-type mice.

Finally, to determine if the effect of eosinophil-deficiency on plasma cell numbers is due to altered local plasma cell proliferation, co-staining for Ki67 and IgA was performed on GIT sections from the small intestine of naïve and *T. gondii*-infected mice by double immunofluorescence. In both naïve and infected ΔdblGATA-1 and wild-type mice, no proliferating IgA^+^ plasma cells (Fig. 6i; IgA^+^Ki67^+^) were observed in any of the stained sections, suggesting that the lamina propria-resident plasma cells do not proliferate *in situ*.

## Discussion

Eosinophils are a major cellular component of the gastrointestinal tract and following parasitic infection the number of eosinophils is significantly increased. While historically being viewed as end-stage effector cells, the eosinophil is increasingly recognised as a cell type that interacts with cells of both the innate and adaptive immune system. For example, the generation and maintenance of IgA^+^ plasma cells is dependent on eosinophils [5, 6, 9]. Previous work has reported a reduction in the number of IgA^+^ plasma cells in both the small and large intestine of eosinophil-deficient mice under steady state conditions [9], although no statistical significance was ascribed to these changes. In keeping with this report we see a non-significant decrease in IgA^+^ plasma cells in the small intestine of eosinophil-deficient mice under steady state conditions, while the number of IgA^+^ plasma cells were equivalent in the uninfected large intestine in the presence or absence of eosinophils.

Importantly, we show for the first time that eosinophils play distinct roles in supporting IgA^+^ plasma cells following infection-driven intestinal inflammation, depending on the gastrointestinal tract niche they occupy. In the large intestine, eosinophil-deficient mice have a significant increase in plasma cells compared to wild-type mice, whereas in the small intestine plasma cell numbers are significantly lower in eosinophil-deficient mice compared to infected wild-type mice. Our data thus demonstrates a relative dependency of plasma cells on eosinophils according to the intestinal niche, and suggests that there is a critical role for eosinophils in maintaining plasma cells in the small but not large intestine. The regional differences in the requirement for eosinophils in maintaining IgA^+^ plasma cells is an important and novel observation, suggesting that the enigmatic functions of the intestinal eosinophil may differ according to intestinal niche, and with this data further emphasising that the two compartments of the gut are distinct and that findings from one should not be extrapolated to the other.

Eosinophils have been identified as a key producer of plasma cell survival factors in the bone marrow [5, 6]. However, it was recently reported that the role of eosinophils in maintaining plasma cells in the intestine appears to be independent of eosinophil-derived APRIL and BAFF [21]. In keeping with this recent publication, our analysis of intestinal tissue revealed no major differences in the plasma cell survival factors APRIL, BAFF, IL-6 or GM-CSF between eosinophil-deficient and control mice following infection. Therefore, although gastrointestinal eosinophils may be capable of producing these survival factors [9], they are not the key source of these factors and other intestinal cells, including epithelial cells [22], T cells and dendritic cells [23] may compensate for the absence of eosinophils. Furthermore, we also assessed whether the differences in IgA^+^ plasma cell frequencies could be attributed to either recruitment or proliferation. Both MAdCAM-1 and CCL25 have previously been suggested to be important in the recruitment of plasma cells to the intestine [19, 20]; therefore the expression of these molecules in eosinophil-deficient and control mice post-infection was analysed. However, our data suggests that differences in MAdCAM-1 or CCL25 expression do not underlie the different phenotypes observed in the small and large intestine in the absence of eosinophils. Moreover, we detected no IgA^+^ plasma cell proliferation within the lamina propria of the small intestine in either the ΔdblGATA-1 or control mice, neither at baseline nor post-infection with *T. gondii*, suggesting that the differences observed in plasma cell numbers is not due to altered proliferation of these cells in the intestinal tissue. This is in agreement with previously published data that shows the majority of plasma cells do not divide within the intestinal lamina propria in C57BL/6 mice [24].

One potential candidate driving differential IgA^+^ phenotypes in the small and large intestine is the microbiota, with the abundance and composition of the microbiota being markedly different in the small versus large intestine [10]. The gut microbiota is known to be important in driving mucosal T cell-independent induction of secretory IgA within the lamina propria [25]. Recently it was reported that the intestinal milieu contributes to the expression of unique niche-dependent transcripts by eosinophils. For example, CD22 is highly expressed on upper GI tract eosinophils, but present at significantly lower levels by eosinophils in other areas of the gut, with colonic eosinophils having the lowest expression levels [12]. Moreover, the duodenum and jejunum are particularly enriched for eosinophils that express ST2, CD69, Ly6C and CD11c [26]. These differences may infer the existence of different subpopulations of eosinophils, with unique functions at different niches throughout the intestine. Therefore, the absence of eosinophils in the small intestine could result in a different effect on plasma cell numbers compared to a deficiency in the large intestinal eosinophil. For example, in niches where eosinophils are rare and appear to be less activated, e.g. expressing lower levels of CD11c, such as the colon [26], there may be a lower dependence on the eosinophil for maintenance of plasma cell numbers with other cell types taking a more prominent role. Again this further emphasises the need for focused regional dissections of immune function along the GIT.

## Conclusion

In conclusion, we show for the first time the impact of eosinophil-deficiency on IgA^+^ plasma cells numbers during inflammation in the small and large intestine. We demonstrate that eosinophils appear to be crucial for the maintenance and/or generation of IgA^+^ cells in the small intestine lamina propria post-infection. In complete contrast, the absence of eosinophils in the inflamed large intestine results in an increase in IgA^+^ cells. Therefore, the role of eosinophils appears to be dependent upon the region of the GIT in which they reside, and this is even more pronounced during inflammation, perhaps reflective of the greater need for restoration of the IgA barrier function.

## Methods

### Animals, *T. muris* and *T. gondii*

BALB/c mice were purchased from Harlan U.K. (Bicester, U.K.). ΔdblGATA-1 mice on a BALB/c background were bred in-house. Male mice were used for all experiments. For *T. muris* infections mice were infected with 20 infective *T. muris* eggs when 8 –10 weeks old and sacrificed at various time points after infection. The maintenance of *T. muris* and the method of infection were as previously described [27], worm burden in the large intestine was assessed as previously described [28]. Tachyzoites of the type I eYFP expressing RH and the tandem dimeric tomato RFP- tagged type II Pruginaud (PRU) strains of *Toxoplasma gondii*, from Boris Striepen [29], were maintained by serial passage through confluent monolayers of human foreskin fibroblasts [30]. Mice were infected by oral gavage with 10^6^ RH or PRU tachyzoites and sacrificed at various time points post infection by exposure to carbon dioxide gas in a rising concentration. In order to ameliorate animal suffering mice were regularly weighed during infection and general appearance monitored, if any mice lost more than 20 % bodyweight they were humanely killed. All animal experiments were approved by the University of Manchester Animal Welfare and Ethical Review Board and performed under the regulation of the Home Office Scientific Procedures Act (1986) and the Home Office approved grant 40/3217.

### Extraction of total RNA and reverse transcription

Tissue samples from the junction of the caecum and large intestine, jejunum or brain were placed in TRIsure (Bioline, London, UK) and frozen on dry ice. Samples were homogenised using a FastPrep 24 and lysing matrix D (MP Biomedicals, Illkirch, France) and total RNA extracted according to the manufacturer’s instructions for TRIsure. Resulting RNA was quantified on a Nanodrop ND-1000 spectrophotometer (Labtech International, East Sussex, U.K.) and stored at −80 °C until used. 1.0 μg of RNA was treated with RNase-free DNase (Promega, Southampton, UK) and reverse transcribed using BioScript (Bioline) in a final volume of 30 μl according to the manufacturer’s instructions and stored at -20 °C.

### Quantitative PCR on intestinal tissue

Quantitative PCR was performed using KAPA SYBR FAST qPCR kit (KapaBiosystems) on a BioRad MyQ^2^ Cycler with Optical System software version 2.1. Housekeeping genes GAPDH and YWAHZ were used as internal controls for gene expression. Expression levels of genes of interest are shown as fold change after normalisation to two housekeeping genes.

**Table:** 

Gene	Forward Primer	Reverse Primer
GAPDH	CCCACTAACATCAAATGGGG	TCTCCATGGTGGTGAAGACA
YWHAZ	TTCTTGATCCCCAATGCTTC	TTCTTGTCATCACCAGCAGC
BAFF	AAGATGGGGAAAGCCGTCAG	CATGGCACACTTCGGTTGTG
APRIL	TCTGTTTGGCTGTGAGGTCA	TCCTGGTCCTCTCGGTCATA
GM-CSF	CTGCGTAATGAGCCAGGAAC	TCAGCGTTTTCAGAGGGCTA
IL-6	GTGGCTAAGGACCAAGACCA	TAACGCACTAGGTTTGCCGA
CCL25	CGCCTCAGACTCTCAGACTGA	CATTGGCACTGGCATGCCTA

### Quantitative PCR for *T. gondii* parasite burden

Parasite burden was assessed as previously described [31]. Briefly, Quantitative PCR was performed using KAPA SYBR FAST qPCR kit (KapaBiosystems) on a BioRad MyQ^2^ Cycler with Optical System software version 2.1. Relative mRNA levels were calculated for toxoplasma cysts (Forward: CGTTTGGAGAAATGGTGTCCCAG; Reverse: CCGCCTGAGTATCCGCTTTTAC) by using an included standard curve for each individual gene and normalised to the housekeeping gene TBP (Forward: AACAGCAGCAGCAACAACAGCAGG; Reverse: TGATAGGGGTCATAGGAGTCATTGG).

### Histology

Histological sections were prepared from proximal large intestine or jejunum and preserved in OCT. 6-μm sections were cut using a microtome and placed on polysine adhesion slides. Immunohistochemical staining for IgA was performed as follows. Slides were fixed in 4 % PFA on ice for 10 mins. Endogenous peroxidase was quenched by incubation with 1.5 U/ml glucose oxidase (Sigma, Gillingham, Dorset, U.K.) in the presence of 1.8 mg/ml glucose and 0.064 mg/ml sodium azide for 20 min at 37 °C. Non-specific binding was blocked with 7 % rat serum (Sigma) and endogenous avidin and biotin binding sites were blocked using a kit according to the manufacturer’s instructions (Invitrogen, Paisley, UK). Sections were then incubated with biotinylated rat anti-mouse IgA (5 μg/ml, BD Biosciences) in phosphate-buffered saline, followed by ABC (avidin-biotin complex) (Vector Laboratories) and 3,3′ Diaminobenzidine (DAB; Vector) and colour development monitored. Sections were counter-stained in HaemQS (Vector Laboratories) for 1 min, and mounted in aquamount (BDH, Lutterworth, UK). For MAdCAM-1 and Siglec F staining the same protocol was used but non-specific binding sites were blocked with 7 % goat serum (Invitrogen), a MAdCAM-1 (5 μg/ml, eBiosciences) or Siglec F primary antibody (5 μg/ml, BD Biosciences) was used followed by a biotinylated secondary goat anti rat Fab’ fragment antibody (1 μg/ml, Santa Cruz). For IgA staining 0.05 % Saponin was utilised in all washing and staining steps.

Slides were blinded and positive cells counted in a minimum of 20 crypts or 10 VCUs from 3 sections evenly distributed across the specimen. Images were acquired using a 20×/0.80 Plan Apo objective using the 3D Histech Pannoramic 250 Flash II slide scanner. Isotype control staining was performed and examined to confirm there was no non-specific staining (Additional file 1: Figure S1).

### Immunofluorescent staining

Histological sections were prepared from proximal large intestine or jejunum and preserved in OCT. 6-μm sections were cut using a microtome and placed on polysine adhesion slides. Slides were fixed in 4 % paraformaldehyde at 4 °C for 10 min. Sections were blocked using the tyramide blocking kit (PerkinElmer, Cambridge, UK) for 30 min. Endogenous biotins were blocked using the avidin/biotin blocking kit as per the manufacturer’s instructions (Invitrogen). Slides were first stained with biotinylated rat anti-mouse IgA (5 μg/ml, BD Biosciences), followed by a secondary avidin Texas-Red (Vector Lab, 30μg/ml). Slides were then incubated with the primary antibody Ki67-Alexa Flour® 488 (5μg/ml, BD Biosciences). Slides were washed and mounted with vector shield containing 4’,6-diamidino-2-phenylindole (Vector Lab). 0.05 % Saponin was utilised in all washing and staining steps.

### Flow cytometry

The proportion of PCs in the MLN was assessed by flow cytometry. Single cell suspensions were incubated with Fc block prior to staining with Biotinylated-B220 or FITC-B220 and APC-CD138 (BD Biosciences) and subsequently Streptavidin-QDOT605 (Invitrogen). Samples were acquired using an LSRII (BD Biosciences) and data were analysed with FACSDiva (BD Biosciences) and FlowJo 10 (Tree Star).

### Statistical analysis

Statistical analysis was performed using the Student’s *t* test or 2 WAY ANOVA with post-hoc Bonferonni’s test, as appropriate with the statistical package GraphPad Prism 6.04 (GraphPad Software, San Diego, U.S.A.). A probability value of <0.05 was considered significant (*p < 0.05, **p < 0.01, ***p < 0.001).

## Abbreviations

ECP, Eosinophil cationic protein; EDN, Eosinophil-derived neurotoxin; EPO, Eosinophil peroxidase; GIT, gastrointestinal tract; MBP, major nasic protein; MLN, mesenteric lymph node; NS, Not significant; PRU, Pruginaud; *T. gondii, Toxoplasma gondii*; *T. muris, Trichuris muris*

## Additional file

Addiotional file 1: Figure S1. Isotype control staining. Representative figures showing isotype control staining in small and large intestine. Scale Bar represents 100 μm. (PDF 2178 kb)
