# Supplementary figures and images for: Eosinophils may play regionally disparate roles in influencing IgA+ plasma cell numbers during large and small intestinal inflammation
Source: BMC Immunol. 2016 May 31;17:12. doi: 10.1186/s12865-016-0153-0 (PMC4886441; doi:10.1186/s12865-016-0153-0)

Small Intestine

Colon

IgA  
isotype  
control

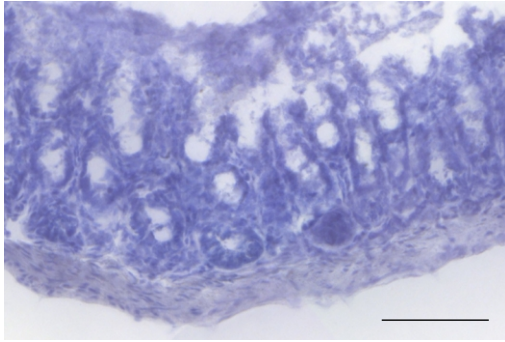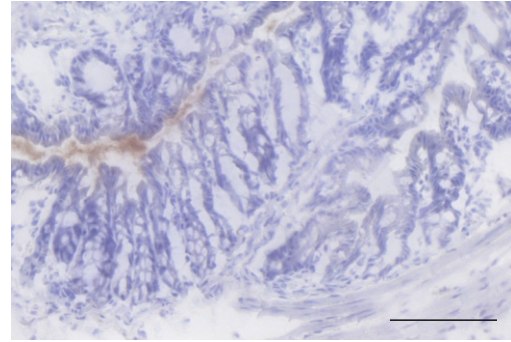

Siglec F  
isotype  
control

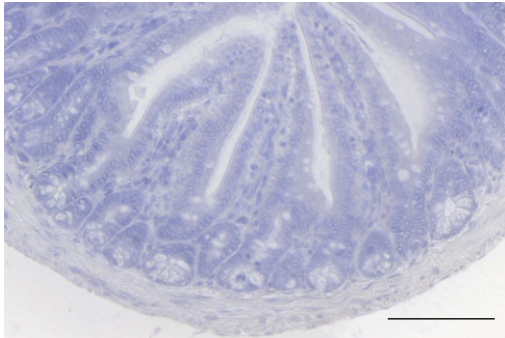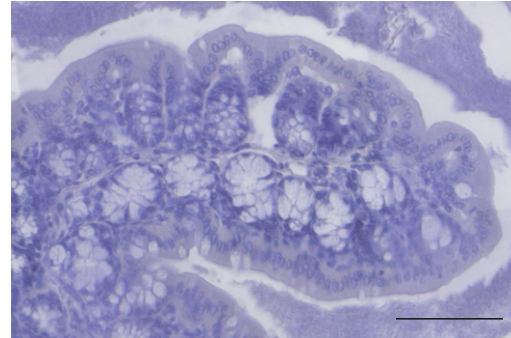

MAdCAM-1  
isotype  
control

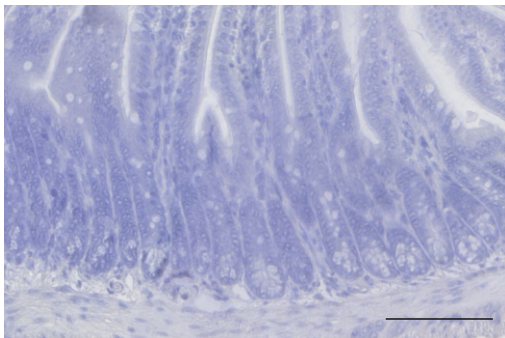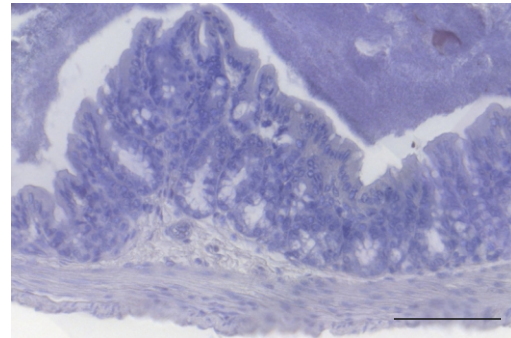

Supplement: Addiotional file 1: Figure S1. — Isotype control staining. Representative figures showing isotype control staining in small and large intestine. Scale Bar represents 100 μm. (PDF 2178 kb) [file 12865_2016_153_MOESM1_ESM.pdf]
